# Supplementary material for: Acute stress reduces effortful prosocial behaviour
Source: eLife. 2024 Jan 5;12:RP87271. doi: 10.7554/eLife.87271 (PMC10942768; doi:10.7554/eLife.87271)
Supplement: Supplementary file 1. — (a) Parameter recovery. We stimulated data based on our trial structure and determined whether the parameters were recoverable for the best-fitting model. There was excellent recovery as showed by the strong relationship between fitted and simulated (or ‘true’) parameters. (b) Spearman’s rho correlations between the difference in how physically demanding participants rated the effort levels after the experiment relative to before and the number of effortful choices in each group for each recipient. p-Values are shown in brackets. There were no significant correlations. (c) Type III Wald test on choice data from the GLMM. The binary dependent variable was choice (0 = rest, 1 = work). Group, Recipient, Effort, Reward, and their interactions were fixed effects. We included a subject-level random intercept and a random slope for Recipient. Significant results are shown in bold. (d) Post hoc comparisons of choice data. The interaction between Group and Effort, and the interaction between Group, Recipient, and Effort, is shown. All p-values are Bonferroni corrected, and significant results are shown in bold. Means were extracted using the emmeans package in R (Lenth, 2022). (e). Type III Wald test on force data from the LMM. Group, Recipient, Effort, Reward, and their interactions were fixed effects, and force was the dependent variable – area under the curve during the force period relative to each participants’ MVC. We included a subject-level random intercept and a random slope for Recipient. Significant results are shown in bold. [file elife-87271-supp1.docx]

**Supplementary file 1a.** Parameter recovery**.** We stimulated data based on our trial structure and determined whether the parameters were recoverable for the best fitting model. There was excellent recovery as showed by the strong relationship between fitted and simulated (or ‘true’) parameters.

| Maximum likelihood correlation | | **Simulated** | | |
| --- | --- | --- | --- | --- |
|  | | *K_self_* | *K_other_* | *β* |
| **Recovered** | *K_self_* | **97.48** | -0.55 | 1.85 |
|  | *K_other_* | -0.48 | **97.50** | 1.73 |
|  | *β* | 12.98 | 13.51 | **77.55** |

**Supplementary file 1b.** Spearman’s Rho correlations between the difference in how physically demanding participants rated the effort levels after the experiment relative to before and the number of effortful choices in each group for each recipient. P values are shown in brackets. There were no significant correlations.

| **Group** | **Self effortful**  **choices** | **Other effortful**  **choices** | **Total effortful**  **choices** | **% Prosocial Choices** |
| --- | --- | --- | --- | --- |
| Control | -0.092  (0.550) | -0.044  (0.775) | -0.119  (0.437) | 0.123  (0.420) |
| Stress | -0.148  (0.321) | 0.043  (0.773) | -0.009  (0.951) | 0.240  (0.104) |
| Both groups | -0.157 (0.135) | -0.065  (0.540) | -0.120  (0.254) | 0.153  (0.146) |

**Supplementary file 1c. Type III Wald test on choice data from the GLMM.** The binary dependent variable was choice (0=rest,1=work). Group, Recipient, Effort, Reward, and their interactions were fixed effects. We included a subject-level random intercept and a random slope for Recipient. Significant results are shown in bold.

|  | **Chi-squared** | **Df** | ***p*-value** |
| --- | --- | --- | --- |
| Group | 1.25 | 1.00 | 0.264 |
| **Recipient** | **29.04** | **1.00** | **<0.001** |
| **Effort** | **895.18** | **4.00** | **<0.001** |
| **Reward** | **928.47** | **4.00** | **<0.001** |
| Group:Recipient | 1.32 | 1.00 | 0.251 |
| **Group:Effort** | **17.27** | **4.00** | **0.002** |
| Recipient:Effort | 5.16 | 4.00 | 0.272 |
| Group:Reward | 5.36 | 4.00 | 0.252 |
| **Recipient:Reward** | **19.12** | **4.00** | **<0.001** |
| Effort:Reward | 19.92 | 16.00 | 0.224 |
| **Group:Recipient:Effort** | **21.48** | **4.00** | **<0.001** |
| Group:Recipient:Reward | 5.03 | 4.00 | 0.285 |
| Group:Effort:Reward | 25.37 | 16.00 | 0.064 |
| **Recipient:Effort:Reward** | **50.65** | **16.00** | **<0.001** |

**Supplementary file 1d. Post-hoc comparisons of choice data.** The interaction between Group and Effort, and the interaction between Group, Recipient and Effort, are shown. All P values are Bonferroni corrected and significant results are shown in bold. Means were extracted using the emmeans package in R (Lenth, 2021).

| **Interaction** | **Comparison** | **Odds ratio** | **95% CIs** | | **Bonferroni-corrected**  ***p-*value** |
| --- | --- | --- | --- | --- | --- |
|  |  |  | **Lower** | **Upper** |  |
| **Group-by-Effort** | Control vs Stress, Effort level 1 | 1.14 | 0.337 | 3.87 | 1.00 |
|  | Control vs Stress, Effort level 2 | 1.30 | 0.394 | 4.30 | 1.00 |
|  | Control vs Stress, Effort level 3 | 3.17 | 0.983 | 10.24 | 0.268 |
|  | Control vs Stress, Effort level 4 | 2.23 | 0.713 | 6.95 | 0.842 |
|  | Control vs Stress, Effort level 5 | 2.37 | 0.763 | 7.34 | 0.679 |
| **Group-by-Recipient-by-Effort** | Group-by-Recipient interaction at effort level 1 | 2.99 | 0.845 | 10.58 | 0.447 |
|  | **Group-by-Recipient interaction at effort level 2** | **4.84** | **1.46** | **15.96** | **0.049** |
|  | Group-by-Recipient interaction at effort level 3 | 1.39 | 0.459 | 4.23 | 1.00 |
|  | Group-by-Recipient interaction at effort level 4 | 1.27 | 0.463 | 3.45 | 1.00 |
|  | Group-by-Recipient interaction at effort level 5 | 0.634 | 0.238 | 1.69 | 1.00 |

**Supplementary file 1e. Type III Wald test on force data from the LMM.** Group, Recipient, Effort, Reward, and their interactions were fixed effects and force was the dependent variable - area under the curve during the force period relative to each participants’ MVC. We included a subject-level random intercept and a random slope for Recipient. Significant results are shown in bold.

|  | **Chi-squared** | **Df** | ***p*-value** |
| --- | --- | --- | --- |
| Group | 0.605 | 1.00 | 0.437 |
| **Recipient** | **16.54** | **1.00** | **<0.001** |
| **Effort** | **27297.55** | **4.00** | **<0.001** |
| **Reward** | **73.40** | **4.00** | **<0.001** |
| Group:Recipient | 0.577 | 1.00 | 0.447 |
| Group:Effort | 8.88 | 4.00 | 0.064 |
| Recipient:Effort | 6.11 | 4.00 | 0.191 |
| Group:Reward | 1.57 | 4.00 | 0.815 |
| Recipient:Reward | 1.55 | 4.00 | 0.818 |
| **Effort:Reward** | **67.92** | **16.00** | **<0.001** |
| Group:Recipient:Effort | 5.43 | 4.00 | 0.246 |
| Group:Recipient:Reward | 4.13 | 4.00 | 0.389 |
| Group:Effort:Reward | 23.93 | 16.00 | 0.091 |
| **Recipient:Effort:Reward** | **46.56** | **16.00** | **<0.001** |
